# Drosophila insulin-like peptide-6 (*dilp6*) expression from fat body extends lifespan and represses secretion of Drosophila insulin-like peptide-2 from the brain

**DOI:** 10.1111/acel.12000

**Published:** 2012-12

**Authors:** Hua Bai, Ping Kang, Marc Tatar

**Affiliations:** Department of Ecology and Evolutionary Biology, Brown UniversityProvidence, RI 02912, USA

**Keywords:** *dilp6*, *dilp2*, insulin/IGF, fat body, fruit fly, longevity

## Abstract

Reduced insulin/IGF signaling extends lifespan in diverse species, including *Drosophila melanogaster* where the genome encodes seven insulin-like peptides (*dilp1-7*). Of these, reduced *dilp2* expressed in the brain has been associated with longevity assurance when over-expression of *dfoxo* in fat bodies extends lifespan. Here, we show that the insulin-regulated transcription factor dFOXO positively modulates *dilp6* mRNA in adult fat body. Over-expression of *dilp6* in adult fat body extends lifespan and increases longevity-associated metabolic phenotypes. Adult fat body *dilp6* expression represses *dilp2* and *dilp5* mRNA in the brain, and the secretion of DILP2 into the hemolymph. The longevity benefit of expressing *dfoxo* in fat body, and the nonautonomous effect of fat body *dfoxo* upon brain dilp expression, is blocked by simultaneously repressing *dilp6* by RNAi in fat body. *dilp6* thus appears to bridge dFOXO, adipose tissue and brain endocrine function to regulate Drosophila longevity.

## Introduction

Insulin-like peptides are evolutionary conserved proteins that regulate growth, metabolism, reproduction, and longevity. Invertebrate genomes are notable for their many insulin-like peptide paralogs. The nematode *Caenorhabditis elegans* has some 40 insulin-like peptides (Pierce *et al*., 2001). Seven insulin-like peptides are encoded in *Drosophila melanogaster* (Drosophila insulin-like peptides, *dilp1-7*), and these are conserved across the genomes of 11 related Drosophilids (Brogiolo *et al*., 2001; Gronke *et al*., 2010). Genes encoding insulin-like peptides have likewise been identified from the silkworm *Bombyx mori* (Bombyxin) and subsequently in orders spanning Orthoptera, Diptera, Lepidoptera, Coleoptera and Hymenoptera (reviewed in (Wu & Brown, 2006)).

The seven Drosophila insulin-like peptides show diverse patterns of stage- and tissue-specific expression (Brogiolo *et al*., 2001; Cao & Brown, 2001; Ikeya *et al*., 2002; Rulifson *et al*., 2002). *dilp2, dilp-4,* and *dilp*-*7* are expressed in the mesoderm and midgut of embryos. *dilp7* mRNA is also present in cells of the larval and adult ventral nerve cord (Brogiolo *et al*., 2001; Yang *et al*., 2008). In larvae, *dilp2*, *dilp-3*, and *dilp*-*5* are predominantly expressed in two clusters of brain neurosecretory cells (IPC, insulin producing cells); these cells are thought to have functional and developmental similarities to mammalian pancreatic β cells (Brogiolo *et al*., 2001; Rulifson *et al*., 2002). Ablating the larval IPC delays metamorphosis, reduces body size and elevates hemolymph carbohydrates (Rulifson *et al*., 2002). In the adult stage, beside its expression in IPC, *dilp5* transcripts were also detected in follicle cells of stage 10 oocytes (Ikeya *et al*., 2002). *dilp3* mRNA is detected in visceral muscle cells of the midgut. *dilp3* expression acts directly on midgut stem cells to regulate intestinal growth (Veenstra *et al*., 2008). *dilp6* is strongly expressed in larval and adult fat body, a tissue with mammalian adipose and liver-like functions. In larvae, the expression of *dilp6* is regulated by dFOXO and is required for pre-metamorphic growth (Okamoto *et al*., 2009; Slaidina *et al*., 2009). Recent studies with larvae revealed that *dilp6* is also expressed in a subset of glia surrounding neuroblasts where this expression leads to neuroblast reactivation upon nutrient restriction (Chell & Brand, 2010; Sousa-Nunes *et al*., 2011).

Insulin-like peptides of the adult help control many traits, including reproduction, metabolism, and lifespan (Hsu & Drummond-Barbosa, 2009; Gronke *et al*., 2010). Reducing insulin/IGF signaling (IIS) increases adult survival (Tatar *et al*., 2003; Giannakou & Partridge, 2007). Lifespan is increased in mutants and dominant-negatives of the Drosophila insulin receptor (*InR*; Tatar *et al*., 2001; Slack *et al*., 2011), and by misexpression of insulin-receptor substrate (*chico*) and PTEN (Clancy *et al*. 2001; Tu *et al*., 2002; Hwangbo *et al*., 2004). Survival is increased when the insulin-producing neurons are ablated (Wessells *et al*., 2004; Broughton *et al*., 2005), which reduces multiple *dilps* as well as any other IPC-related neuropeptides. It has proved more difficult to analyze the impact of individual *dilps* on lifespan because the seven related genes exhibit compensatory expression (Broughton *et al*., 2008; Min *et al*., 2008). Nonetheless, analysis of homologous recombination knockouts of individual *dilp* genes revealed that loss of *dilp2* was sufficient to increase survival (Gronke *et al*., 2010).

A role for *dilp2* in the control of aging was also suggested by studies that extended lifespan through the IIS-related factors dFOXO (Hwangbo *et al*., 2004), Jun-N-terminal kinase (JNK; Wang *et al*., 2005), and short neuropeptide-F (sNPF, homolog of mammalian NPY; Lee *et al*., 2008). Work with dFOXO is notable because lifespan was extended when this IIS-regulated transcription factor was over-expressed in adult fat body in a diet-dependent manner (Min *et al*., 2008). Lifespan was extended and *dilp2* mRNA was reduced in adults fed a low-yeast diet when *dfoxo* was over-expressed from abdominal fat body. In contrast, lifespan was extended and *dilp2* was reduced in flies fed a high-yeast diet when *dfoxo* was over-expressed from head fat body. In both conditions, systemic IIS signaling in peripheral tissues was reduced, while *dilp2* mRNA of the IPC was less abundant.

From this view, FOXO plays both autonomous and non-autonomous roles in aging. Work with the fly heart illustrates the autonomous role where increased dFOXO specifically within cardiac tissue is sufficient to slow heart functional aging (Wessells *et al*., 2004). dFOXO is proposed to mediate feedback signaling between the IPC and fat bodies because *dilp* from the IPC could repress dFOXO within fat body while fat body dFOXO regulates *dilp* expression in the IPC. Besides non-autonomously modulating the expression of *dilp* mRNA in the adult brain, dFOXO has been reported to regulate the transcription of *dilp6* within fat body, at least in the case of larvae (Okamoto *et al*., 2009; Slaidina *et al*., 2009). In larvae, dFOXO is required for starvation-activated *dilp6* expression where dFOXO protein binds to the promoter region of the *dilp6* locus. This observation stimulated the questions for the current work: Is *dilp6* regulated by dFOXO in adult fat body, does *dilp6* expressed from the adult fat body modulate aging, and if so, might it do so by regulating the expression and secretion of *dilps* produced by the adult IPC, especially *dilp2*?

To address these issues, we over-expressed or silenced *dilp6* in specific adult tissues including fat body. Previous work that systemically and ubiquitously knocked out genomic *dilp6* reported this to decrease larval growth but not to effect adult survival (Gronke *et al*., 2010). Here, we show that *dilp6* over-expressed in adult fat body extends lifespan, elevates carbohydrate and fat storage, and improves oxidative stress resistance. *4ebp* mRNA, a transcriptional target of dFOXO, is elevated in tissues aside from the site of *dilp6* over-expression, suggesting that over-expression of fat body *dilp6* systemically reduced insulin/IGF signaling. In these conditions, *dilp2* and *dilp5* mRNA of the brain were repressed, and hemolymph DILP2 was significantly reduced as measured by an enzyme immunoassay. *dilp6* RNAi in fat body blocks the longevity benefit typically observed when *dfoxo* is over-expressed in fat body, and this *dilp6* RNAi likewise blocks the negative effect of fat body expressed *dfoxo* upon *dilp2* mRNA. Fat body *dilp*6 forms part of the non-autonomous aging-control circuit between dFOXO, fat body, and brain.

## Results

### *dilp6* is expressed in adult adipose tissue and is induced by fasting and dFOXO

**Fig. 1:**
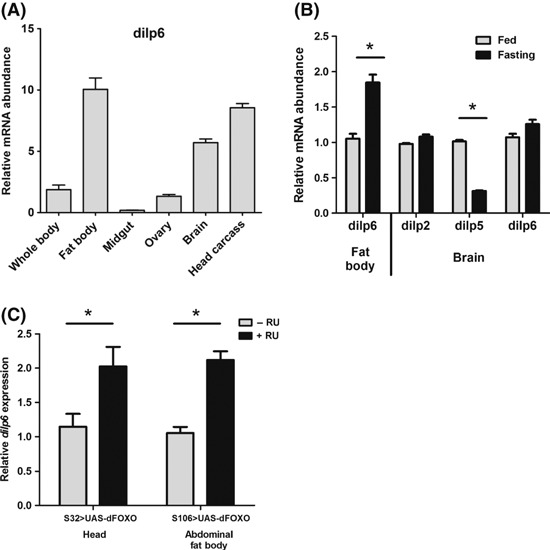
*dilp6* tissue distribution and response to fasting. (A) *dilp6* mRNA measured from fat body, midgut, ovary, brain and head carcass. (B) *dilp* mRNA measured from overnight fasted females: in fat body *dilp6* increased upon fasting, in brain, only *dilp5* is reduced upon fasting. (C) *dfoxo* over-expressed (UAS–*dfoxo*-TM) in fat body (S32: head; S106 abdominal) up-regulates *dilp6* mRNA. Asterisk indicates significant difference between treatment and control (*P* < 0.05). RU: RU486 (mifepristone).

To date, *dilp6* function has been best described in larval fat body (Okamoto *et al*., 2009; Slaidina *et al*., 2009). To study *dilp6* in the adult, we measured its expression in several tissues (Fig. 1A). *dilp6* mRNA is apparent in abdominal fat body, brain and head carcass (head fat body, compound eyes, antennae, and mouth parts) but is relative rare in midgut and ovary. This contrasts with *dilp1, dilp2, dilp3, and dilp5*, which are predominantly expressed in the brain at the central IPC (Fig. S1). *dilp6* expression in abdominal fat body and head fat body is also observed from a *dilp6*-GAL4 insertion line (NP1079) driving UAS-*GFP.nls*, while in the brain, the expression pattern of *dilp6* is distinct from primary insulin producing neurons (IPC; Fig. S2).

Slaidina *et al*. (2009) reports *dilp6* mRNA in larval fat body was strongly induced by fasting. Here, we see *dilp6* mRNA up-regulated in abdominal fat body of overnight fasted adults, while brain *dilp5* mRNA was repressed and *dilp2* mRNA was static (Fig. 1B). Unlike its expression in fat body, *dilp6* mRNA in the brain does not change upon fasting (Fig. 1B). In late-stage larvae, fasting activates dFOXO and this induces transcription of *dilp6* (Slaidina *et al*., 2009). To test whether such control operates in adults, we drove a constitutively active dFOXO (UAS–*dfoxo*-TM (Hwangbo *et al*., 2004)) with the fat body-specific, RU486 inducible (GeneSwitch, GS) S_1_32-Gal4 (head fat body), and S_1_106-Gal4 (abdominal fat body; Roman *et al*., 2001). Over-expression of *dfoxo* in either head fat body (via S32-Gal4) or abdominal fat body (via S106-Gal4) up-regulated endogenous *dilp6* mRNA expression in head and abdominal fat body, respectively (Fig. 1C).

### Over-expressing *dilp6* in adipose tissue extends lifespan

**Fig. 2:**
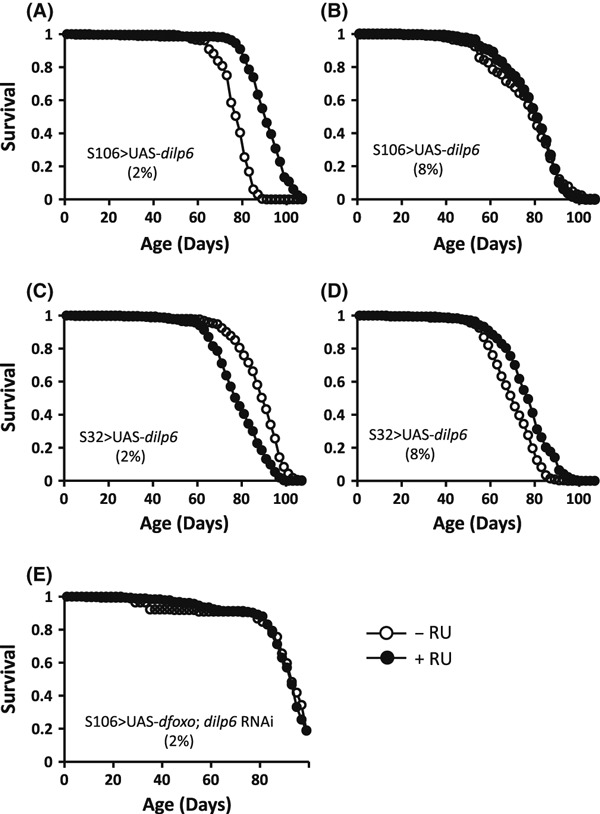
*dilp6* expressed in fat body extends lifespan. (A) *dilp6* over-expressed in abdominal fat body (via S106-GS-Gal4) extends lifespan in flies maintained on low-yeast diet, but not (B) when maintained on high-yeast diet. (C) *dilp6* over-expressed in head fat body (via S32-GS-Gal4) does not extend lifespan on low-yeast diet but (D) moderately extends lifespan on high-yeast diet. (E) Simultaneous induction of UAS-*dfoxo* and UAS-*dilp6* RNAi inhibits the survival benefit expected from over-expressing *dfoxo* alone in fat body.

As a target of dFOXO in adult fat body, *dilp6* may mediate longevity assurance conferred by over-expressing *dfoxo*. Consistent with this prediction, conditional expression of *dilp6* in head and abdominal fat body extended female lifespan (Table 1). Notably, *dilp6* from abdominal fat body extended lifespan (Fig. 2A) and consistently reduced age-specific mortality (Fig. S11A) in females maintained upon relatively low-yeast diet (2% yeast) but not on high-yeast diet (8% yeast; Fig. 2B). This pattern of diet dependence is similar to the nutrient conditions when *dfoxo* over-expression in abdominal fat body extends lifespan (Min *et al*., 2008). Likewise, *dilp6* conditionally expressed in head fat body modestly increased lifespan in females upon high-yeast diet (Fig. 2D,F) and less so upon low-yeast diet (Fig. 2C). No detectable effect of *dilp6* upon lifespan was seen for males on any diet or when expressed from either fat body (Table 1). In contrast to these results, lifespan was shortened by conditional expression of *dilp6* with ubiquitous drivers, as well as when *dilp6* was ubiquitously reduced by RNAi (Table 1; Figs S3 and S4). Lifespan was not affected when *dilp6* was over-expressed by a conditional pan-neuronal driver or when silenced by RNAi in fat body (Table 1; Figs S3 and S4). As *dfoxo* over-expressed in fat body can extend lifespan (Hwangbo *et al*., 2004; Giannakou *et al*., 2005), we tested whether reducing *dilp6* is sufficient to block the longevity benefit of the *dfoxo* transgene. As expected if *dilp*6 modulates the longevity benefit of dFOXO from the fat body, there was no survival or mortality differences between control and RU-induced cohorts of the genotype S_1_106-Gal4 > UAS-*dfoxo*; UAS-*dilp6* (RNAi; Figs 2E and S11C).

**Table 1:** Median lifespan of adult flies with *dilp6* over-expression and knockdown

				Median lifespan (*E*_0_, days)				
							
Sex	GS-Gal4	UAS	Diet % yeast	0 RU	200 RU	*E*_0_ dif. (%)	*P*	Sample size (no. flies)
Male	S32-GS	UAS-*dilp6*	2%	81	77	−4.94	<0.0001	727
Male	S32-GS	UAS-*dilp6*	8%	75	77	2.67	0.37	693
Male	S106-GS	UAS-*dilp6*	2%	77	79	2.60	0.8714	582
Male	S106-GS	UAS-*dilp6*	8%	73	67	−8.22	<0.0001	593
Female	S32-GS	UAS-*dilp6*	2%	89	77	−13.48	<0.0001	700
Female	S32-GS	UAS-*dilp6*	8%	69	75	8.70	<0.0001	662
Female	S106-GS	UAS-*dilp6*	2%	77	89	15.58	<0.0001	573
Female	S106-GS	UAS-*dilp6*	8%	79	79	0.00	0.9667	578
Female	Tub-GS	UAS-*dilp6*	4%	79	53	−32.91	<0.0001	748
Female	da-GS	UAS-*dilp6*	4%	97	87	−10.31	<0.0001	741
Female	Elav-GS	UAS-*dilp6*	4%	67	73	8.96	0.1159	688
Female	Tub-GS	*dilp6* RNAi	4%	85	75	−11.76	<0.0001	749
Female	S32-GS	*dilp6* RNAi	4%	71	71	0.00	0.179	714
Female	S106-GS	*dilp6* RNAi	4%	77	75	−2.60	<0.0001	650

Diets contained cornmeal, sugar, agar and either 2%, 4% or 8% yeast.

Probability is based on chi-square distribution from log-rank test between control (0 RU) and induced (200 RU) cohorts. GeneSwitch-Gal4 (GS) divers were used for *dilp6* over-expression: S32-GS (head fat body), S106-GS (abdominal fat body), *Tub*-GS (ubiquitous), *da*-GS (ubiquitous), Elav-GS (pan-neuronal).

### *dilp6* regulates adult metabolism, stress resistance, and fecundity

**Fig. 3:**
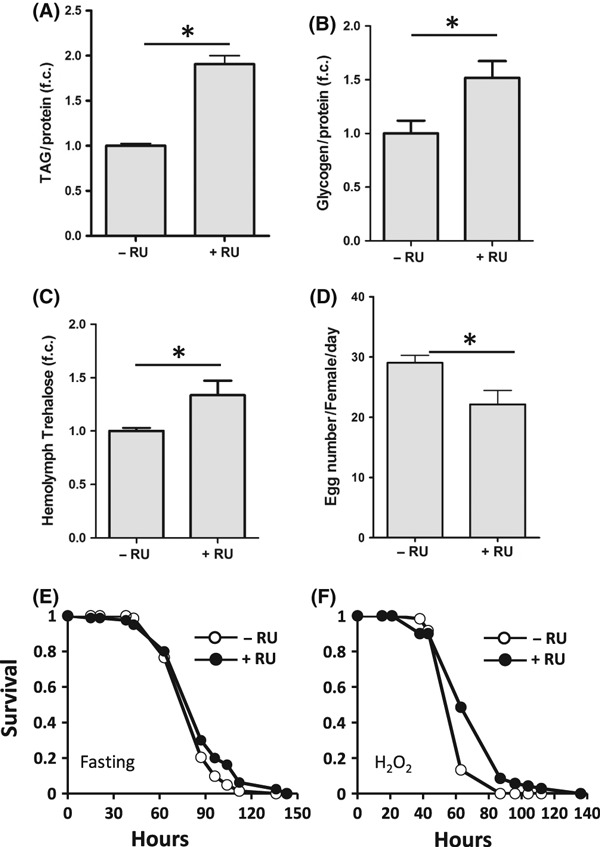
*dilp6* expressed in abdominal fat body modulates metabolism, fecundity and stress resistance. *dilp6* over-expressed in abdominal fat body increases fat body (A) triglycerides, (B) glycogen, and (C) trehalose in hemolymph, while (D) repressing fecundity. Asterisk indicates significant difference between treatment and control, *P* < 0.05. *dilp6* over-expressed in abdominal fat body modestly increases the resistance (E) to starvation (log-rank test, *P* < 0.048, *n* = 150) and (F) to H_2_O_2_ (*P* < 0.0003, *n* = 130).

**Fig. 4:**
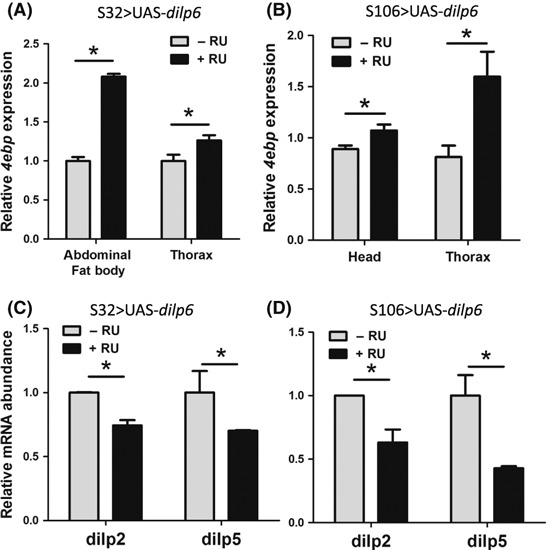
*dilp6* expressed in fat body reduces systemic insulin/IGF signaling. *4ebp* mRNA is increased in abdominal fat body and thorax when *dilp6* is over-expressed in head fat body (A), while *4ebp* message is increased in the head and thorax when *dilp6* is over-expressed in abdominal fat body (B). *dilp2* and *dilp5* mRNA measured from the head are reduced when *dilp6* is over-expressed in head fat body (C) and abdominal fat body (D). Asterisk indicates significant difference between treatment and control (*P* < 0.05).

Manipulations that systemically reduce insulin/IGF signaling and extend Drosophila lifespan often induce metabolic and stress-resistant phenotypes. These stereotypic traits were seen when *dilp6* was expressed from adult fat body. This increased whole body triacylglycerides (TAG) and glycogen, and hemolymph trehalose (Fig. 3A–C). Nutrient storage is often associated with survival during fasting but only a modest fasting survival benefit was observed when *dilp6* was conditionally expressed from fat body (Fig. 3E). These females, however, exhibited elevated survival when challenged with H_2_O_2_ oxidative stress (Fig. 3F). Fecundity was slightly reduced when *dilp6* was expressed from fat body (Fig. 4D). These patterns of stress resistance, metabolite storage, and extended lifespan suggest that increased expression of *dilp6* may coordinate phenotypes by reducing insulin production from the brain.

### Fat body *dilp*6 modulates neuronal *dilp2* mRNA and secreted *dilp*2 protein

**Fig. 5:**
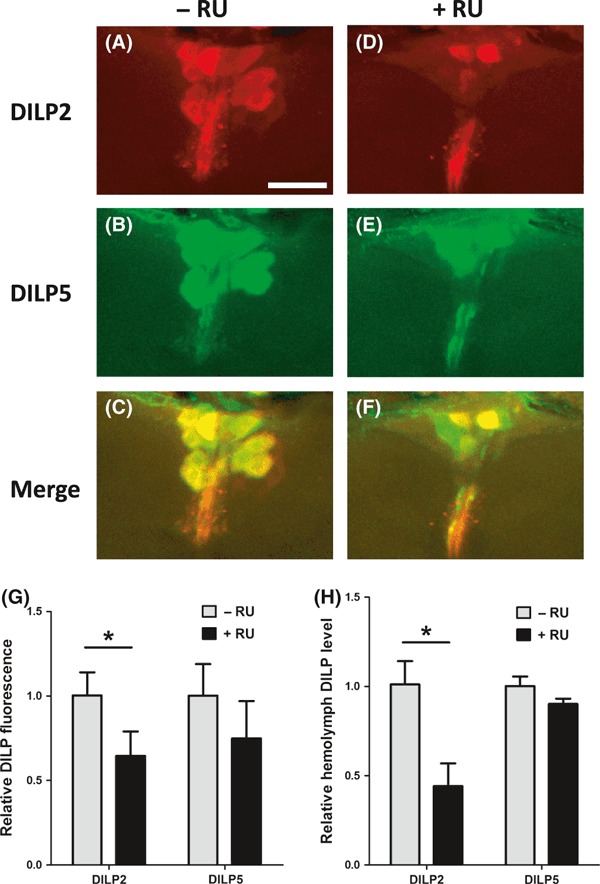
Fat body *dilp6* reduces *dilp2* mRNA and *dilp*2 secretion. (A–F) The cellular distribution of *dilp*2 (red) and *dilp*5 (green) changes within IPC when *dilp6* is over-expressed in abdominal fat body. (G) Quantified immunostaining fluorescence of *dilp*2 in the IPC is significantly reduced in *dilp6* over-expressing flies. Mean ± SE of eight replicate preparations. (H) EIA assay for *dilp* peptides in hemolymph. Circulating DILP2 is significantly reduced when *dilp6* is over-expressed in abdominal fat body. Asterisk indicates significant difference between treatment and control (*P* < 0.05). Scale bar: 20 μm.

**Fig. 6:**
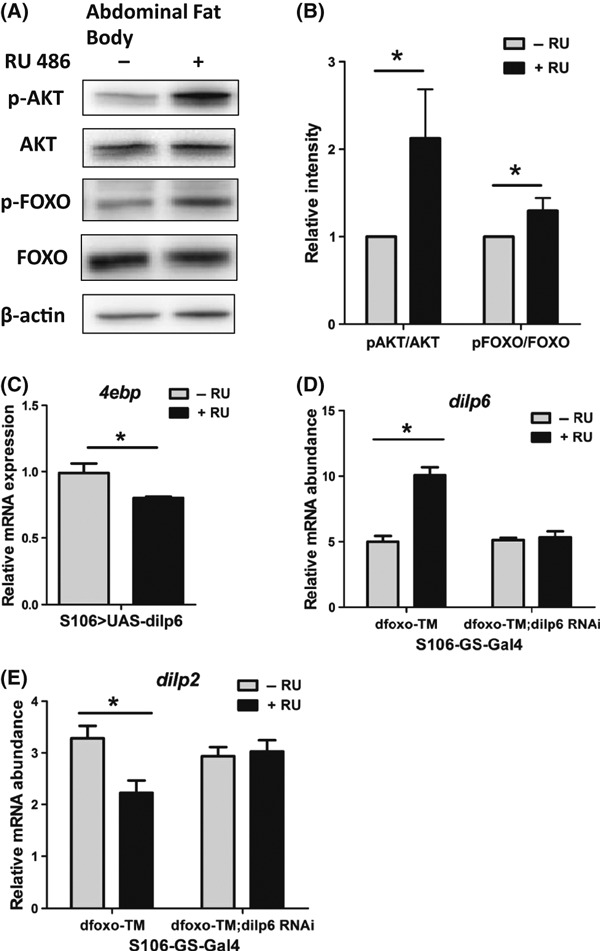
*dilp*6 is a local insulin/IGF agonist and required for non-autonomous effects of *dfoxo* upon brain insulin/IGF expression. (A) Western blot: phospho-Akt and phospho-FOXO in abdominal fat body are increased when *dilp6* is over-expressed in this tissue, quantified in (B; *n* = 4). (C) *4ebp* mRNA in abdominal fat body is reduced when *dilp6* was over-expressed in this tissue. Asterisk indicates significant difference between treatment and control (*P* < 0.05). Simultaneous expression of *dfoxo* and *dilp6*(RNAi) in fat body (D) prevents blocks the normal over-expression of *dilp6* induced by *dfoxo* and (E) prevents the repression of *dilp2* mRNA at a distance in the brain.

The transcription factor *4ebp* is a direct transcriptional target of dFOXO that is induced when insulin signaling is repressed (Puig *et al*., 2003). Here, *4ebp* mRNA was upregulated in abdominal fat body and thorax when *dilp6* was over-expressed in head fat body (Fig. 4A), while *4ebp* message was increased in head tissues when *dilp6* was over-expressed in abdominal fat body (Fig. 4B). These effects at a distance from the site of *dilp6* manipulation suggest that peripheral insulin/IGF may be reduced in *dilp6* over-expression flies, and *dilp2* and *dilp5* mRNAs were indeed reduced in brains from females where *dilp6* was over-expressed in fat body (Figs. 4C,D). At the same time, *dilp*2 peptides in IPC bodies were significantly reduced (Fig. 5A–C,G), while *dilp*5 peptides were only modestly affected (Fig 5D–G). To determine whether *dilp6* of fat body represses the secretion of *dilp* protein, we measured hemolymph DILP titer by enzyme immunoassay (EIA) using antibodies against DILP2 or DILP5. When driven in abdominal fat body, *dilp6* over-expression strongly reduced the level of circulating DILP2 but this manipulation only modestly affected DILP5 (Fig. 5H). Furthermore, the ability of *dfoxo* expressed in the fat body to regulate the expression and secretion of DILP in the brain requires fat body *dilp6*. Simultaneously, driving UAS-*dfoxo* and UAS-*dilp6*(RNAi) in fat body precludes the elevation of *dilp6* mRNA in this tissue (Fig. 6D) and prevents the expected reduction of brain *dilp2* mRNA when UAS*-dfoxo* alone is driven in fat body (Fig. 6E).

Reduced hemolymph DILP2 is likely to produce the observed reduction in IIS signaling of peripheral tissues. However, these responses could also arise if DILP6 from the fat body acts an insulin-receptor antagonist. To address this alternative, we measured the phosphorylation of Akt and FOXO in abdominal fat body at the site where *dilp6* was over-expressed. Both p-Akt and p-FOXO were elevated in fat body that over-expressed *dilp6* (Fig. 6A,B). Furthermore, *4ebp* transcripts were repressed when *dilp6* was over-expressed in the same tissue (Fig. 6C). DILP6 of fat body thus appears to act as a local insulin/IGF agonist. DILP6 appears to indirectly repress insulin/IGF signaling in other tissues by non-autonomously reducing DILP2 secretion from the brain.

## Discussion

The Drosophila genome contains one insulin-receptor gene with several isoforms, and seven *dilp* loci (Brogiolo *et al*., 2001). Among these *dilps*, reduced *dilp2* has been consistently associated with increased lifespan. Homologous recombination to knockout *dilp2* increased longevity (Gronke *et al*., 2010), and mRNA levels of *dilp2* were reduced and longevity was increased in genotypes that misexpressed JNK (Wang *et al*., 2005), sNPF (Lee *et al*., 2008), and *dfoxo* (Hwangbo *et al*., 2004; Giannakou *et al*., 2005) where *dfoxo* misexpression from fat body alone was sufficient to slow aging. The targets of dFOXO responsible for its non-autonomous control of aging are unknown, and there are potentially many candidates. dFOXO can bind to at least 700 promoter regions (Alic *et al*., 2011), and this factor has been associated with the transcriptional control of more than 1000 genes (Zinke *et al*., 2002; Gershman *et al*., 2007). At least in larvae, *dilp6* is a target of dFOXO and through its expression in fat body *dilp6* modulates growth during post-feeding development (Okamoto *et al*., 2009; Slaidina *et al*., 2009).

The present study finds expression of *dfoxo* in adult fat body is sufficient to increase local *dilp6* expression, and this decreases systemic insulin signaling. Decreased systemic insulin signaling is not likely to arise because *dilp*6 acts as an antagonist, unlike as has been suggested for *ins-1* of *C. elegans* (Pierce *et al*., 2001). In the fly, *dilp6* expressed within fat body increases insulin-receptor-mediated phospho-signaling and decreases *4ebp* mRNA of the fat body, indicating *dilp6* is a local insulin signaling agonist. Rather, decreased systemic insulin is associated with a 50% reduction in circulating DILP2 peptide. Reduced circulating DILP2 may account for the observed increase in lifespan when *dilp6* is expressed in fat body, and for the correlated changes in storage lipids and carbohydrates, oxidative stress resistance, and fecundity.

dFOXO control of *dilp6* and the subsequent effects of *dilp6* upon DILP secreted from IPC may contribute to an insulin-regulatory feedback loop between fat body and insulin producing neurons of the brain (Fig. S10). dFOXO expressed in adult fat body represses *dilp2* mRNA in the IPC (Hwangbo *et al*., 2004). When DILP2 secretion is reduced, the fat body will further induce dFOXO and thus reinforce the repression of *dilp2* in the IPC. This circuit will reinforce a state of low circulating DILP and thus promote longevity assurance. Events that stimulate DILP synthesis in the IPC could shift the feedback to an alternative stable state with active DILP secretion. Elevated DILP secretion would thus suppress fat body dFOXO and thus release the dFOXO suppression of *dilp* expression in the IPC. *dilp6* may play an intermediary role in this regulatory circuit because it is a target of fat body dFOXO, while it is also upstream of *dilp* expression and secretion in the IPC.

The factors transmitting signals from *dilp*6 of fat body to the IPC of the brain are unknown. Fat body-derived DILP6 itself may be secreted and function as this hormone. Alternatively, *dilp*6 may regulate a downstream adipokine that circulates to affect the brain IPC, or it may affect the brain through changes in systemic metabolism to which the IPC are sensitive. In general, the roles and functions of the adult fat body of Drosophila are enigmatic. This tissue shares functions found in mammalian liver as well as adipose tissue. Drosophila fat body participates in immune function (antimicrobial peptide expression), yolk protein synthesis, and energy homeostasis and storage, although metabolic data are mostly known from studies of larvae (Colombani *et al*., 2003; Okamoto *et al*., 2009; Slaidina *et al*., 2009). In larvae, the amino acid transporter Slimfast and TOR function in the fat body as nutrient sensors to coordinate systemic growth (Colombani *et al*., 2003). On the basis of data from co-culture experiments, amino acid-restricted fat body secretes a hormonal factor (an adipokine) to remotely control DILP secretion from IPC (Geminard *et al*., 2009), and this process requires TOR signaling in the fat body. Whether TOR signaling from adult fat body has the capacity to regulate lifespan has not been satisfactorily resolved (*erratum* to Kapahi *et al*., 2004).

The potential for adipokines to modulate longevity assurance may be conserved across taxa. In mammals, systemic insulin signaling is influenced by fat body-derived hormonal factors, such as tumor necrosis factor-alpha (TNF-α), leptin and adiponectin. Inflammatory cytokine TNF-α and TNF-α receptor knockout mice present increased insulin sensitivity (Schreyer *et al*., 1998). Leptin, first observed in mutant obese mice, regulates food intake by acting on hypothalamic neuropeptide Y signaling (Friedman & Halaas, 1998). In pancreatic islets and β cells culture, leptin inhibits glucose and glucagon-like peptide-1 stimulated insulin secretion (Zhao *et al*., 1998). Although the Drosophila genome reveals no obvious homolog of leptin, over-expression of Drosophila sNPF, an ortholog of mammalian neuropeptide Y, increases adult *dilp1* and *dilp2* mRNA, and conversely, knock-down of sNPF decreases these *dilps* and extends lifespan (Lee *et al*., 2008). Notably, *dilp6* expressed from adult abdominal fat body reduces expression of sNPF from the brain (Fig. S7), and this reduction may contribute to the suppression of *dilp2* in the IPC (Lee *et al*., 2008). In addition to leptin, mammalian adiponectin secreted from adipose tissue profoundly affects insulin resistance and type 2 diabetes (Kadowaki *et al*., 2006). Transgenic mice expressing human adiponectin in the liver have increased circulating adiponectin, reduced fasting glucose, insulin, and leptin and improved survival when fed high-fat/high-sucrose diet (Otabe *et al*., 2007). Intriguingly, elevated adiponectin and polymorphisms of the adiponectin gene (*ADIPOQ*) are enriched in human centenarians (Atzmon *et al*., 2008). Drosophila contains a predicted homolog of the adiponectin 1 receptor (CG5315) that has cell-autonomous and non-autonomous roles in regulating oogenesis in response to diet (LaFever, 2010), but an adiponectin-like ligand has yet to be identified.

In Drosophila and mammals, there are reciprocal interactions between fat body and systemic insulin signaling. Fat body can produce adipokines to modulate systemic IIS sensitivity and stimulation, while insulin signaling within the fat body itself responds to these systemic insulin changes. Identifying the roles and functions of adipokines in Drosophila may provide an avenue to understand central regulatory mechanisms of aging and its interaction with metabolism.

## Experimental procedures

### Fly stocks and husbandry

RU486 (RU, mifepristone)-induced drivers (all referred to here as GeneSwitch, GS) were as follows: *Tub-*GS*-Gal4; Elav-*GS*-Gal4* (Roman *et al*., 2001); *da-GS-Gal4* (Tricoire *et al*., 2009); *S32-*GS*-Gal4*; *S106-*GS*-Gal4* (Roman *et al*., 2001); *dilp2-GS-Gal4* (kindly provided by Dr. Heinrich Jasper). *dilp6-Gal4* (#103877, DGRC-Kyoto stock center) is a constitutive driver. Transgenes responding to Gal4 were as follows: UAS-*GFP.nls* (gift from Bruce Edgar, Heidelberg, Germany); UAS-*dfoxo-TM* and UAS-*dfoxo* (Hwangbo *et al*., 2004); UAS-*dilp6* (Slaidina *et al*., 2009); *UAS*-*dilp6* (RNAi) (#31379, Bloomington stock center). Expression and knock-down efficiency of UAS-*dilp6* and UAS-*dilp6*(RNAi) are presented in Fig. S6 (Supporting information). Data in Figs S8 and S9 (Supporting information) demonstrate that *S106-*GS*-Gal4* does not induce transgene expression in the absence of RU486 and that RU486 alone has no side effects upon egg production, TAG, *dilp2* or *dilp5* expression or adult survival.

Flies were reared and maintained at 25 °C, 40% relative humidity and 12-h light/dark. Adults were maintained upon agar-based diet with cornmeal (0.8%), sugar (10%), and yeast (Lesaffre Yeast Corp., Milwaukee, WI, USA; 2% or 8% as specified). RU486 (mifepristone; Sigma, St. Louis, MO, USA) to activate GeneSwitch-Gal4 was dissolved in ethanol and added to the food at a concentration of 200 μm. Fasting was performed overnight by transferring flies to glass vials with 1% agar media.

### Quantitative RT–PCR

Total RNA was extracted from 10 whole flies or from tissue of 15 flies in Trizol reagent (Invitrogen, Grand Island, NY, USA). DNase-treated total RNA was quantified with a NanoDrop ND-1000 (Thermo Fisher Scientific Inc., Wilmington, DE, USA). About 50–100 ng of total RNA was used for quantification with SuperScript™ One-Step RT-PCR reagent (Invitrogen) and measured on an ABI prism 7300 Sequence Detection System (Applied Biosystems, Carlsbad, CA, USA), with three biological replicates for each experimental treatment. mRNA abundance of each gene was normalized to ribosomal protein L32 (*RpL32*) by the method of comparative *C*_T_.

### Demography and survival analysis

Two- to three-day-old adult flies were collected with CO_2_ anesthesia and pooled in 1 L demography cages at a density of 100–125 flies per cage, with three independent cages per genotype. Food vials with media containing vehicle only or RU486 were changed every 2 days, at which time dead flies were removed and recorded. Survival analysis was conducted with JMP statistical software with data from replicate cages combined. Survival distributions were compared by the log-rank test.

### TAG, glycogen, and trehalose

Triglycerides (TAG) and glycogen were quantified from abdominal fat body dissected from 10 female adults and homogenized in lysis buffer (0.2% Tween-20 in PBS). The lysate was heated for 5 min at 70 °C and then centrifuged at 14 000 ***g*** for 5 min. TAG was measured from 10 μL of supernatant with Infinity Triglycerides Reagent (Thermo Fisher Scientific Inc, Wilmington, DE, USA). Protein was quantified via BCA Protein Assay (Pierce, Thermo Fisher Scientific Inc./Pierce, Rockford, IL, USA). To measure glycogen, 30 μL of the supernatant was incubated with amyloglucosidase (Carolina Biological, Burlington, NC, USA) for 30 min at 37 °C to convert glycogen to glucose; glucose was measured with Infinity Glucose Reagent (Thermo Fisher Scientific Inc).

To measure trehalose, hemolymph was collected from 20 centrifuged, decapitated flies (Broughton *et al*., 2008; Demontis & Perrimon, 2010). 0.3–0.5 μL of hemolymph was diluted in PBS, and the glucose concentration was measured with Infinity Glucose Reagent (Thermo Fisher Scientific Inc) after incubation with porcine trehalase (Sigma) at 37 °C overnight.

### Female fecundity

Three-day-old mated female flies were maintained on food with or without RU486 for 5 days at one female per vial and 10–15 vials per group. Flies were daily passed to new vials over 3 days (with or without RU486), and eggs were counted daily. RU486 alone had no effect on fecundity as shown in previous studies (Slack *et al*., 2011) and Fig. S9 (Supporting information).

### Stress resistance

Starvation resistance was measured in 5-day-old females previously maintained on food with and without RU486. Females were transferred into glass vials containing 1% agar with or without RU486. Dead flies were counted twice a day. To assess oxidative stress resistance, 5-day-old females previously maintained on food with and without RU486 were transferred into glass vials containing 1% agar, 5% sucrose, and 5% H_2_O_2_ (with or without RU486). Dead flies were counted twice a day. In both assays, ∼80 females distributed among 8 vials were tested in each group. Effects on survival were analyzed by the log-rank test.

### Antibodies and immunostaining

Antibodies included anti-DILP2 (1:200), anti-DILP5 (1:200; Geminard *et al*., 2009); anti-rat IgG-Alexa Fluor 594 (1:300); and anti-rabbit IgG-DyLight 488 (1:300; Jackson ImmunoResearch, West Grove, PA, USA). Samples were processed as described in Geminard *et al*. (2009) and imaged with a Leica SP2 laser scanning confocal microscope. To quantify fluorescence signaling, confocal Z-stack images were obtained with identical laser power and scan settings (Leica Microsystems Inc., Buffalo Grove, IL, USA). The integrated density for IPC was measured and subtracted from the density of background readings using ImageJ software.

### Enzyme immunoassay (EIA) for hemolymph *dilp*

About 0.5 μL of hemolymph was diluted with PBS and incubated overnight in cells of a 96-well EIA/RIA plate (Corning Incorporated, Corning, NY, USA) at room temperature. Following incubation, cells were cleared of hemolymph, and bound material in the plate was blocked for 2 h with EIA buffer (10 mm Na_2_HPO_4_, 3 mm NaH_2_PO_4_, 150 mm NaCl, 1 mm NaEDTA · 2dH_2_O, 0.2% Na azide) and 1% BSA. Blocked samples were washed three times with PBS-Tween. Except for the blank well, samples were treated with 100 μL of anti-DILP2 or anti-DILP5 antibody at 1:2500 dilution, incubated 1 h at room temperature, washed three times with PBS-Tween, and treated with HRP-conjugated secondary antibody (1:2500). In the final step, the plate was washed and treated with TMB solution (3,3′,5,5′-teramethylbenzidine; American Qualex antibodies, San Clemente, CA, USA) to provide colorimetric quantification. This reaction was stopped by 100 μL 1 m phosphoric acid, and absorbance was recorded at 450 nm upon a Molecular Device M5 reader (Sunnyvale, CA, USA). Serial dilutions of synthetic DILP2 (provided by Dr. James Wade, University of Melbourne) and recombinant DILP5 (Sajid *et al*., 2011) were used to determine specificity and quantitative response of these EIA (Fig. S5). Both assays provided specific and linear response ranges for peptide concentrations of 1–10 ng.

### Western blot

Antibodies for Akt, Phospho-Drosophila Akt (Ser505), Phospho-FOXO1 (Ser256) and β-actin were purchased from Cell Signaling Technology. Drosophila FOXO antibody was generated against the peptide sequence corresponding to amino acids 1–233 and affinity purified. Abdominal fat bodies were homogenized in RIPA buffer with protease inhibitor cocktail (Sigma). Supernatant was incubated with SDS loading buffer (Invitrogen) at 70 °C for 10 min. About 30 μg of denatured protein was separated on 10% SDS-polyacrylamide precast gels (Invitrogen) and transferred to nitrocellulose membranes. Following incubation with primary and secondary antibodies, the blots were visualized with Pierce ECL Western Blotting Substrate (Thermo Fisher Scientific Inc). Band intensity was quantified with AlphaView software (Bio-Rad Laboratories Inc., Hercules, CA, USA).

## Supporting Information

**Fig. S1** *dilp1, dilp2, dilp3* and *dilp5* mRNA measured from fat body, midgut, ovary, brain and head carcass.

**Fig. S2** DistribuLon of *dilp6* reporter in adipose tissue and brain.

**Fig. S3** Lifespan of *dilp6* over-expression using ubiquitous drivers: (A) *Tub-GeneSwitch-Gal4*, (B) *da-GeneSwitch-Gal4*, (C) neuronal *Elav-GeneSwitch-Gal4*.

**Fig. S4** Lifespan of *dilp6* silencing using ubiquitous drivers: (A) *Tub-GeneSwitch-Gal4*; and fat body (B)*S32-GeneSwitch-Gal4*, (C)*S106-GeneSwitch-Gal4*.

**Fig. S5** Specificity of anL-DILP2 and anL-DILP5 anLbodies in EIA.

**Fig. S6** Upon 2% and 8% yeast diet, qRT-PCR verifies the inducLon of *dilp6* by *UAS-dilp6* (A,B), and *dilp6* knock-down by *RNAi* (C).

**Fig. S7** sNPF transcripts measured in flies with ubiquitous and Lssue-specific *dilp6 overexpression*.

**Fig. S8** S106-gal4 does not induce transgene expression without RU.

**Fig. S9** RU486 alone does not induce aging or metabolic phenotypes.

**Fig. S10** Model for DILP6 to regulate lifespan by repressing DILP produced in the brain.

**Fig. S11** Mortality rate (estimated as −ln(ln(px)) for survival plots of text figure 2.
