# Supplementary material for: Drosophila insulin-like peptide-6 (dilp6) expression from fat body extends lifespan and represses secretion of Drosophila insulin-like peptide-2 from the brain
Source: Aging Cell. 2012 Dec;11(6):978–85. doi: 10.1111/acel.12000 (PMC3500397; doi:10.1111/acel.12000)

Supplemental Figures

Drosophila insulin-like peptide-6 (*dilp6*) expression from fat body extends lifespan and represses secretion of Drosophila insulin-like peptide-2 from the brain

Hua Bai, Ping Kang, Marc Tatar

Supplemental Figure 1. *dilp1, dilp2, dilp3* and *dilp5* mRNA measured from fat body, midgut, ovary, brain and head carcass. All flies were age 7d, Org[R] females. Mean and stand error of three biological replicates.

Supplemental Figure 2. Distribution of *dilp6 reporter in adipose tissue and brain. A dilp6-GAL4 enhancer trap insertion line (NP1079) was crossed to UAS-GFP.nls. dilp6 is promoted in abdominal fat body (A) and head fat body (B). (C) DILP6 expression is also seen in several brain regions distinct from IPC of the pars intecebralis that produce dilp1, 2, 3, 5. Arrow indicate location of pars intecebralis IPC. Scale bar: 20 µm.*

Supplemental Figure 3. Lifespan of *dilp6 over-expression using ubiquitous drivers: (A) Tub-GeneSwitch-Gal4, (B) da- GeneSwitch-Gal4, (C) neuronal Elav- GeneSwitch-Gal4.*

Supplemental Figure 4. Lifespan of *dilp6 silencing using ubiquitous drivers: (A) Tub-GeneSwitch-Gal4; and fat body (B) S32-GeneSwitch-Gal4, (C) S106-GeneSwitch-Gal4.*

Supplemental Figure 5. Specificity of anti-DILP2 and anti-DILP5 antibodies in EIA. (A) Serial dilution of DILP2 peptide (0.625 ng, 1.25 ng, 2.5 ng, 5 ng and 10 ng) produced a linear response with anti-DILP2 but no response with anti-DILP5. (B) Serial dilution of DILP5 peptide (1.25 ng, 2.5 ng, 5 ng, 10 ng and 20 ng) produced a linear response with anti-DILP5 but no response with anti-DILP2.

Supplemental Figure 6. Upon 2% and 8% yeast diet, qRT-PCR verifies the induction of *dilp6 by UAS-dilp6 (A,B), and dilp6 knock-down by RNAi (C).*

Supplemental Figure 7. sNPF transcripts measured in flies with ubiquitous and tissue-specific *dilp6 over-expression. Ubiquitous driver: Tub-GS; fat body drivers: S106-GS and S32-GS; pan-neuronal driver: Elav-GS.*

Supplemental Figure 8. S106-gal4 does not induce transgene expression without RU. Left column panels are a positive control where In larval fat body the constitutive tissue pumpless-Gal4 (ppl-Gal4) drives GFP expression. In the right columns, without RU the GeneSwitch S106-Gal4 does not drive the same UAS-GFP.

Supplemental Figure 9. RU486 alone does not induce aging or metabolic phenotypes. Flies with the S106-GS-Gal4 driver but no UAS-transgene were fed RU at our standard concentration (200 uM). This treatment alone failed to affect fecundity, TAG, dilp expression or survival.

Supplemental Figure 10. Model for DILP6 to regulate lifespan by repressing DILP produced in the brain. Altered insulin/dFOXO signaling in fat body cells modulates *dilp6 expression, which in turn directly or indirectly regulates brain DILP secretion. Lifespan is modulated by dFOXO through cell autonomous in tissues responding to reduced systemic DILP2.*

Supplemental Figure 11. Mortality rate (estimated as $-\ln(\ln(p_x))$ for survival plots of text figure 2. Plots A and B demonstrate that expression dilp6 in fat body consistently reduces age-specific mortality ($1-p_x$) and thus extends lifespan by reducing demographic senescence. Plot C shows overlap of mortality rate between control flies and the cohort simultaneously expressing dfoxo and dilp6(RNAi); as suggested by their survival plot (figure 2 E), there are no differences in demographic aging between these groups.

Supplemental Figure. 1

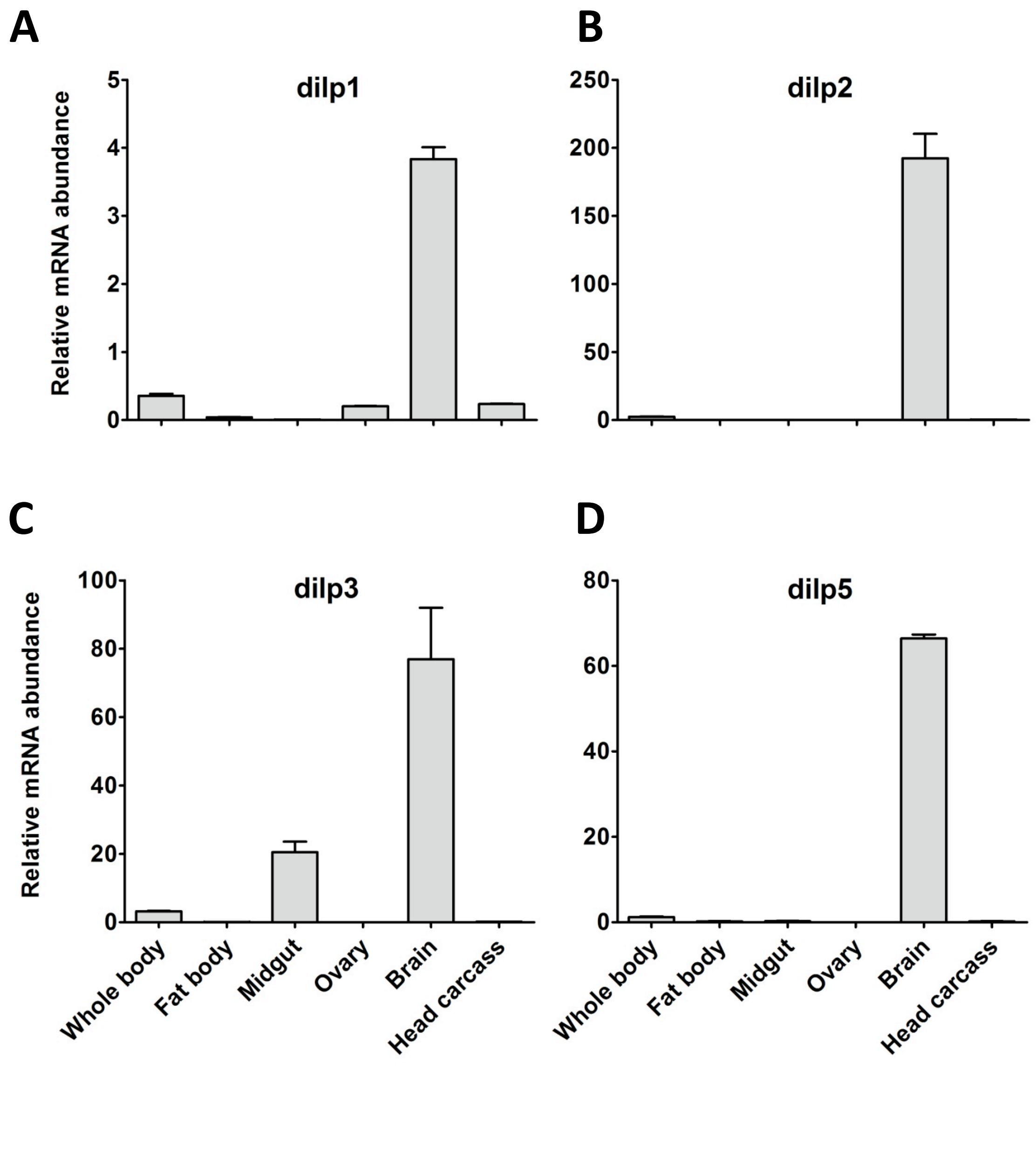

Supplemental Figure. 2

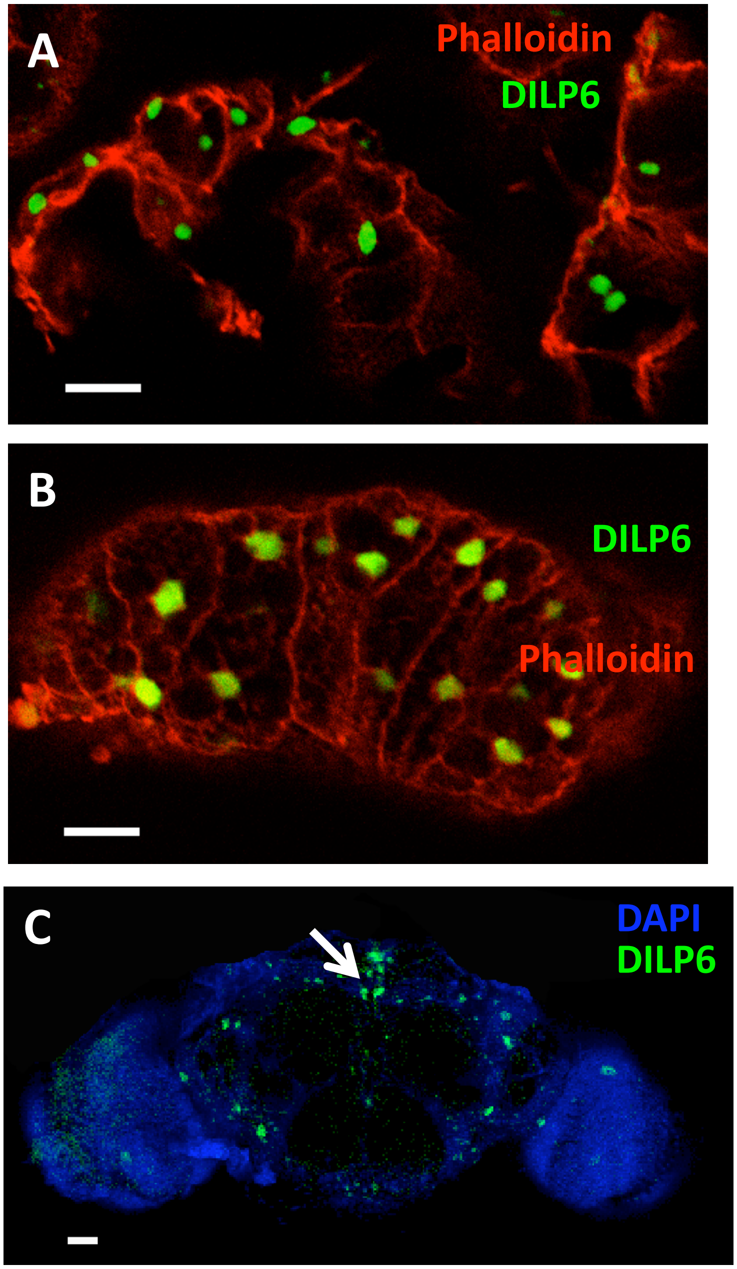

Supplemental Figure. 3

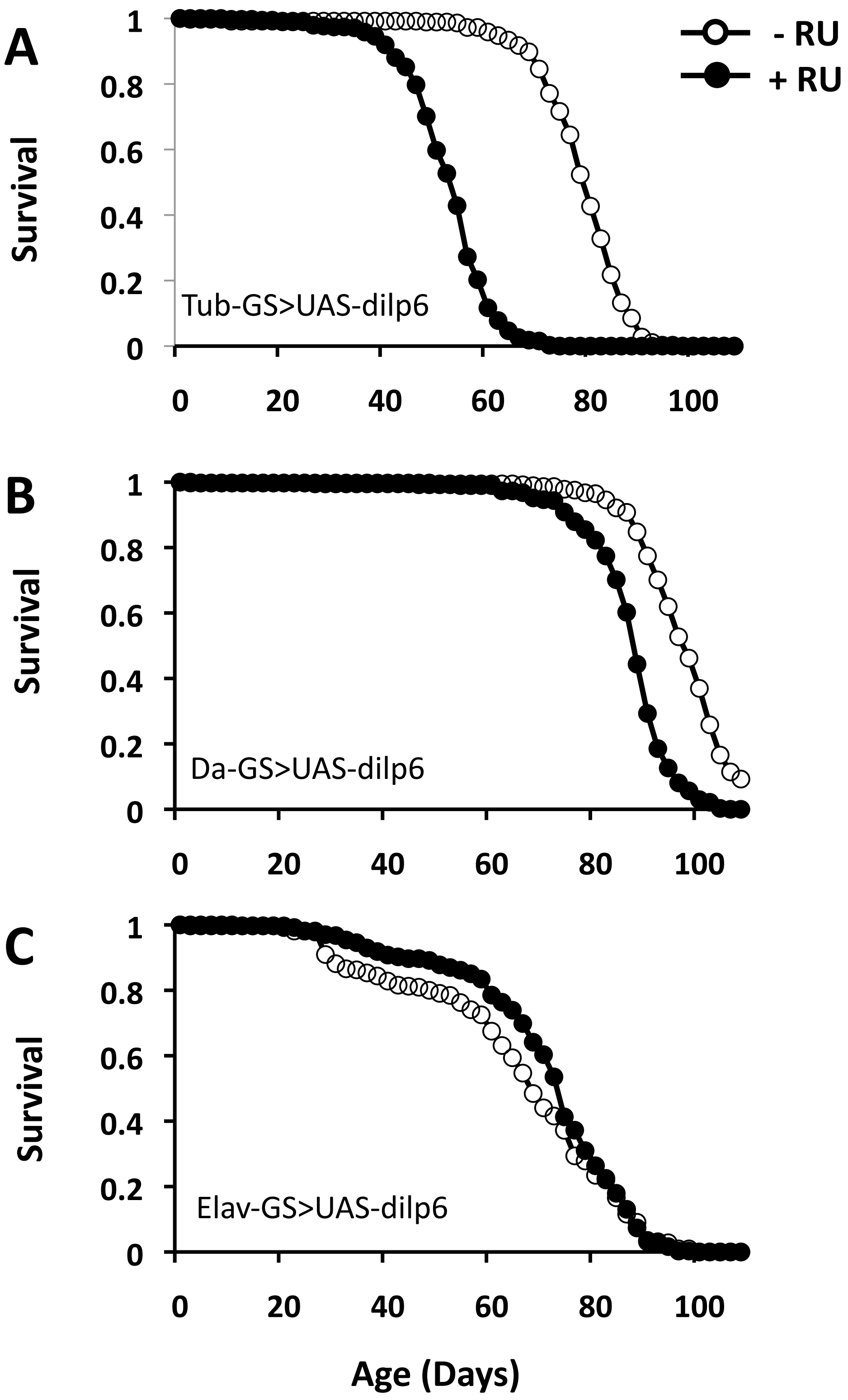

Supplemental Figure. 4

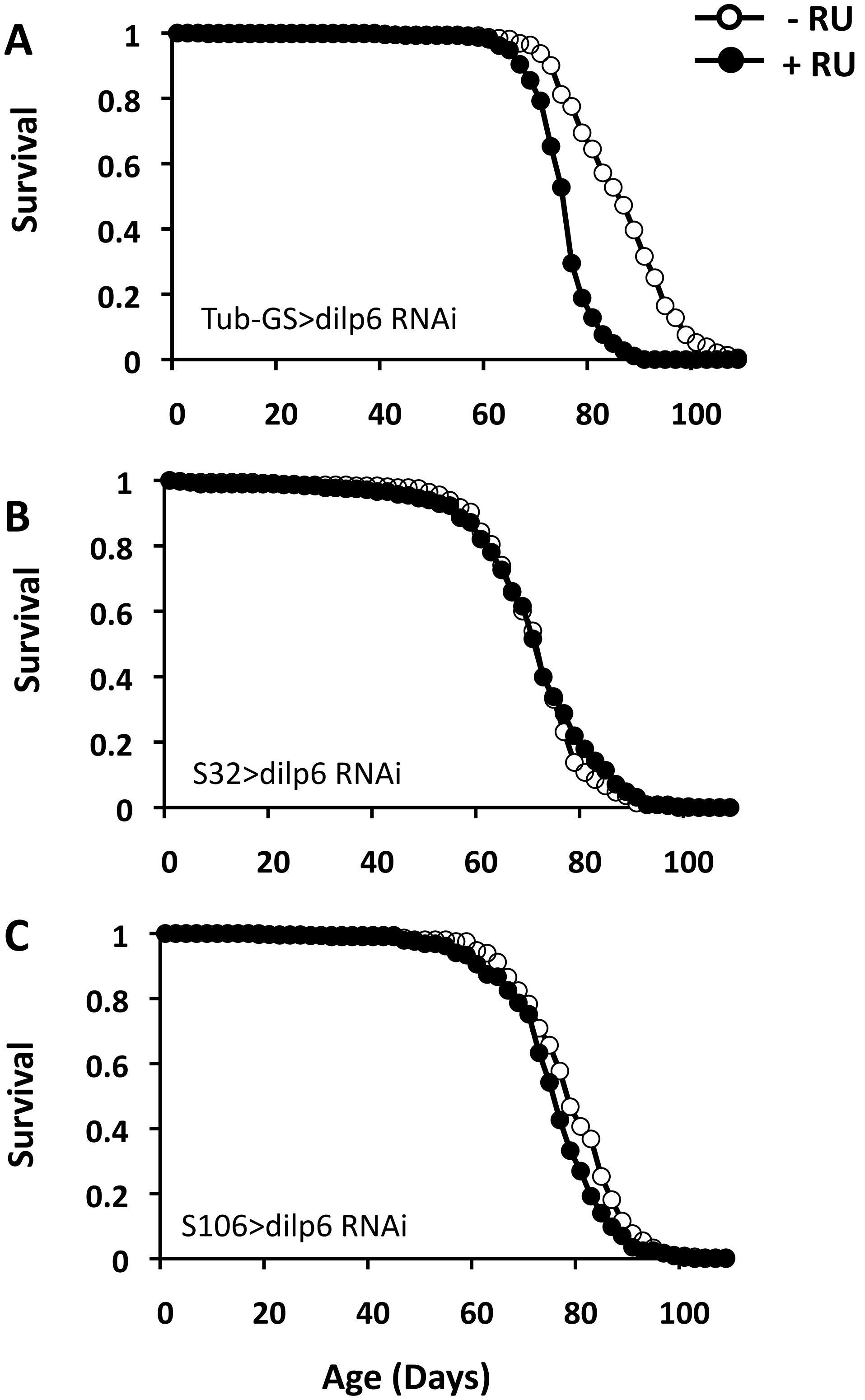

Supplemental Figure. 5

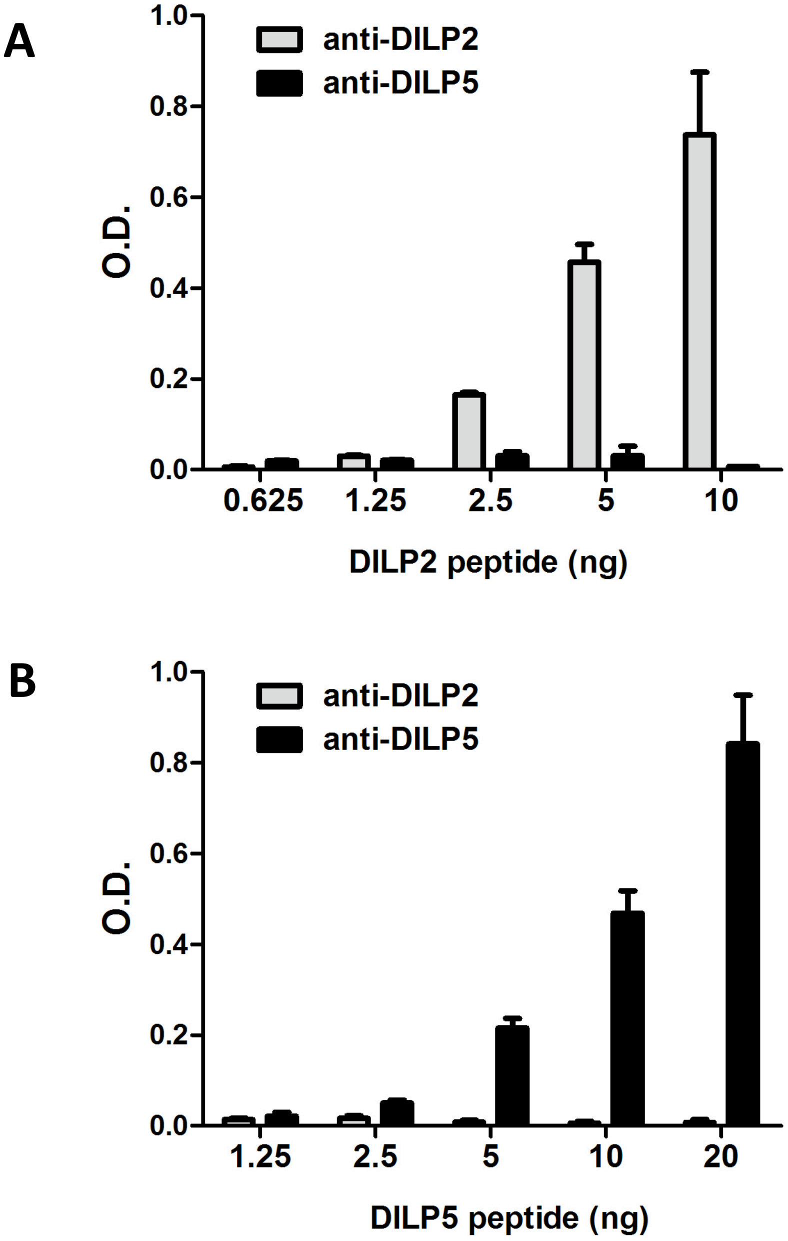

Supplemental Figure. 6

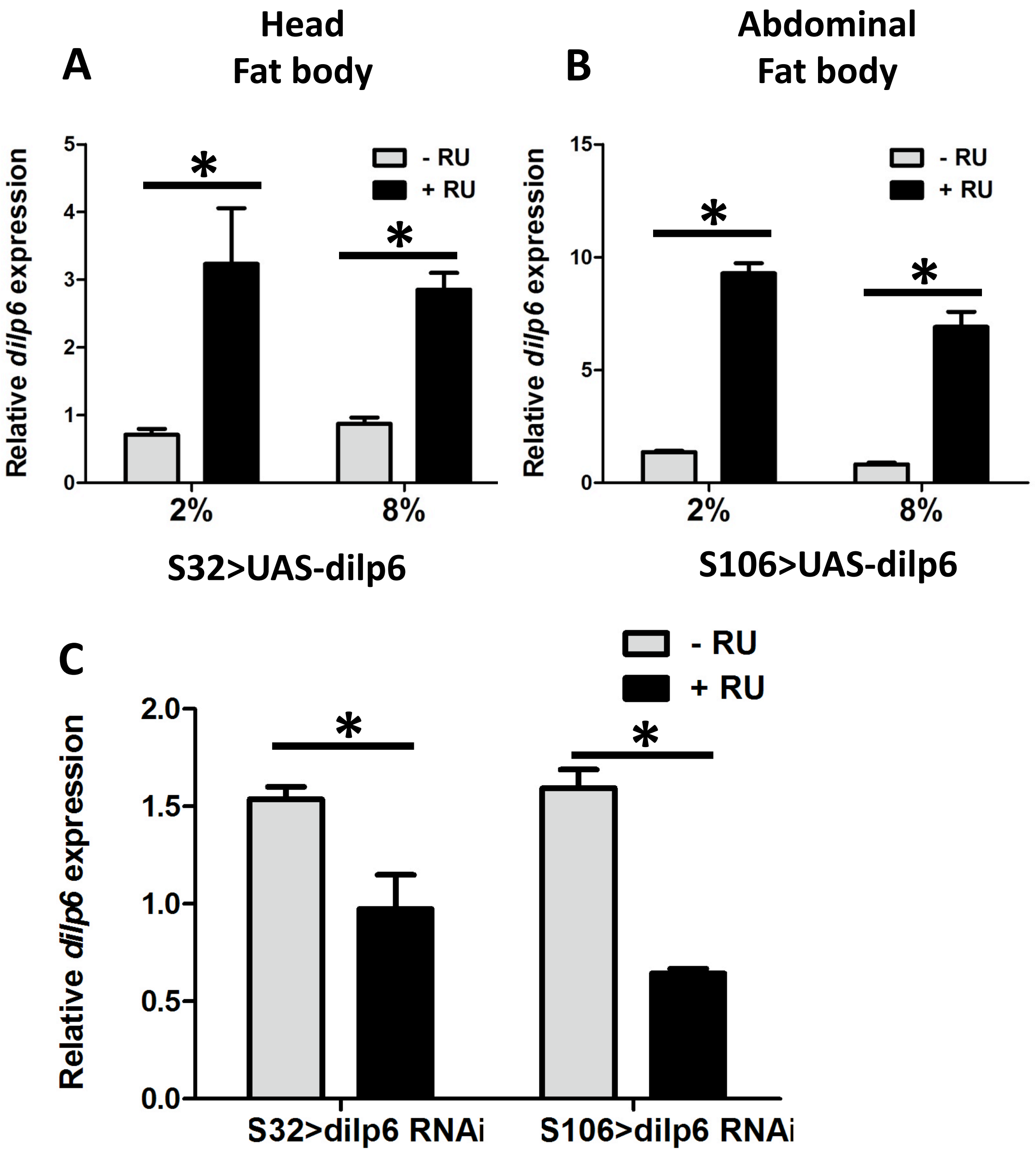

Supplemental Figure. 7

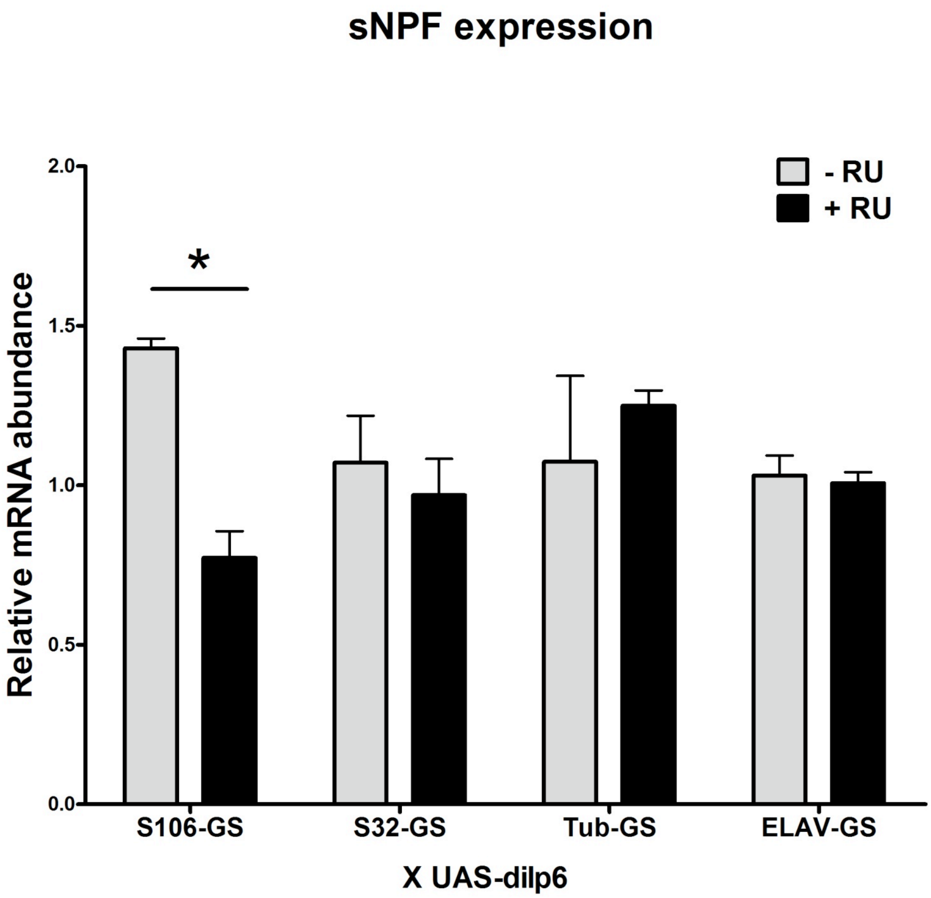

Supplemental Figure. 8

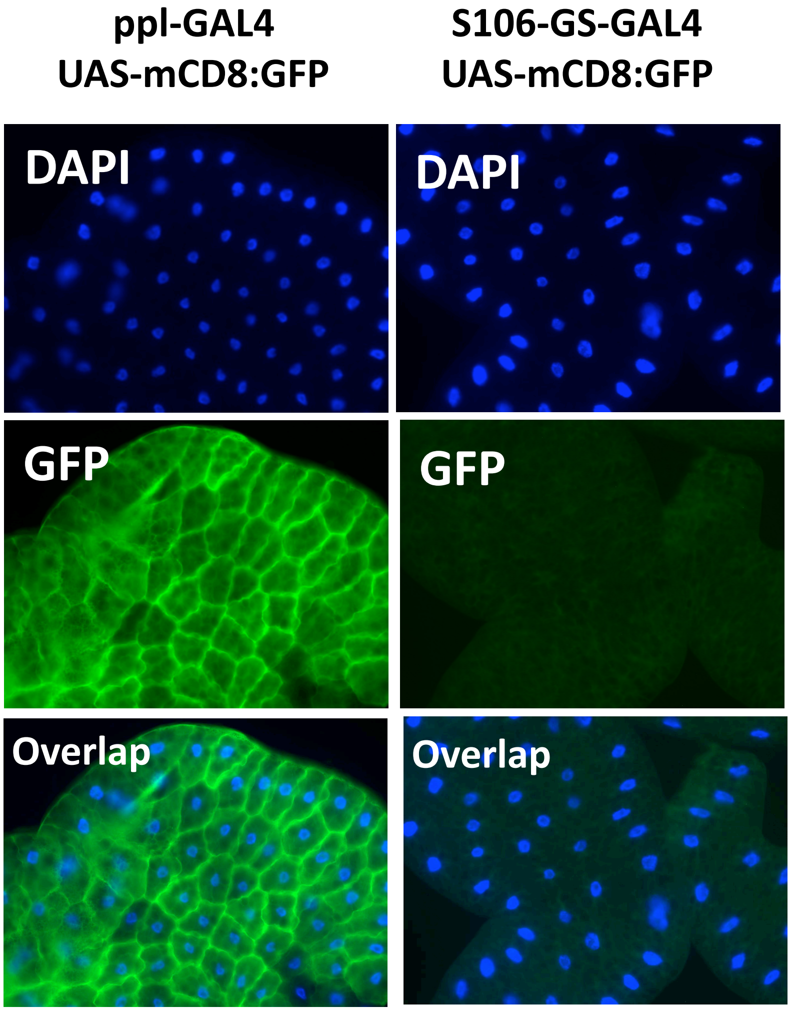

(Larval fat body)

Supplemental Figure. 9

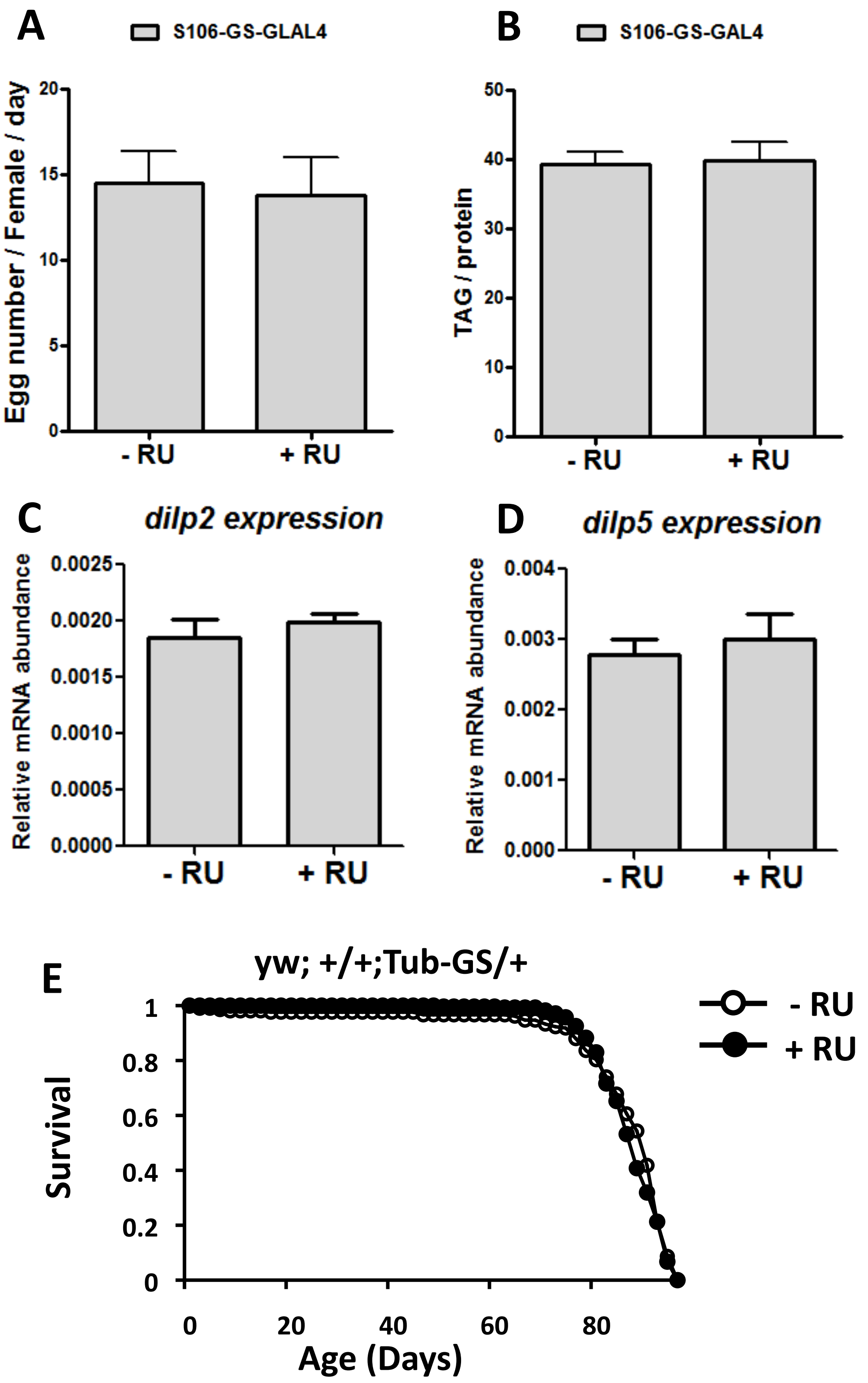

Supplemental Figure. 10

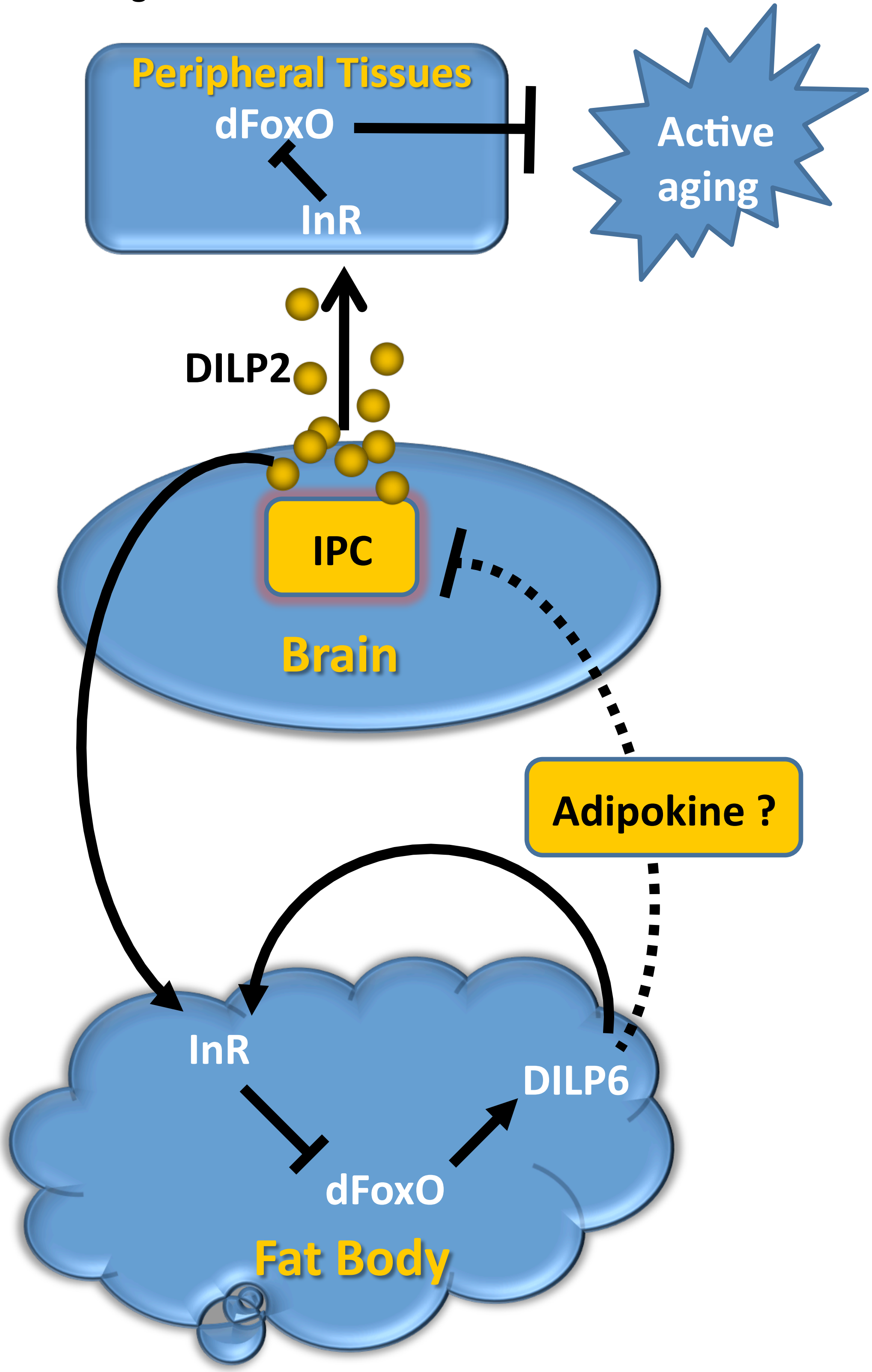

Supplemental Figure. 11

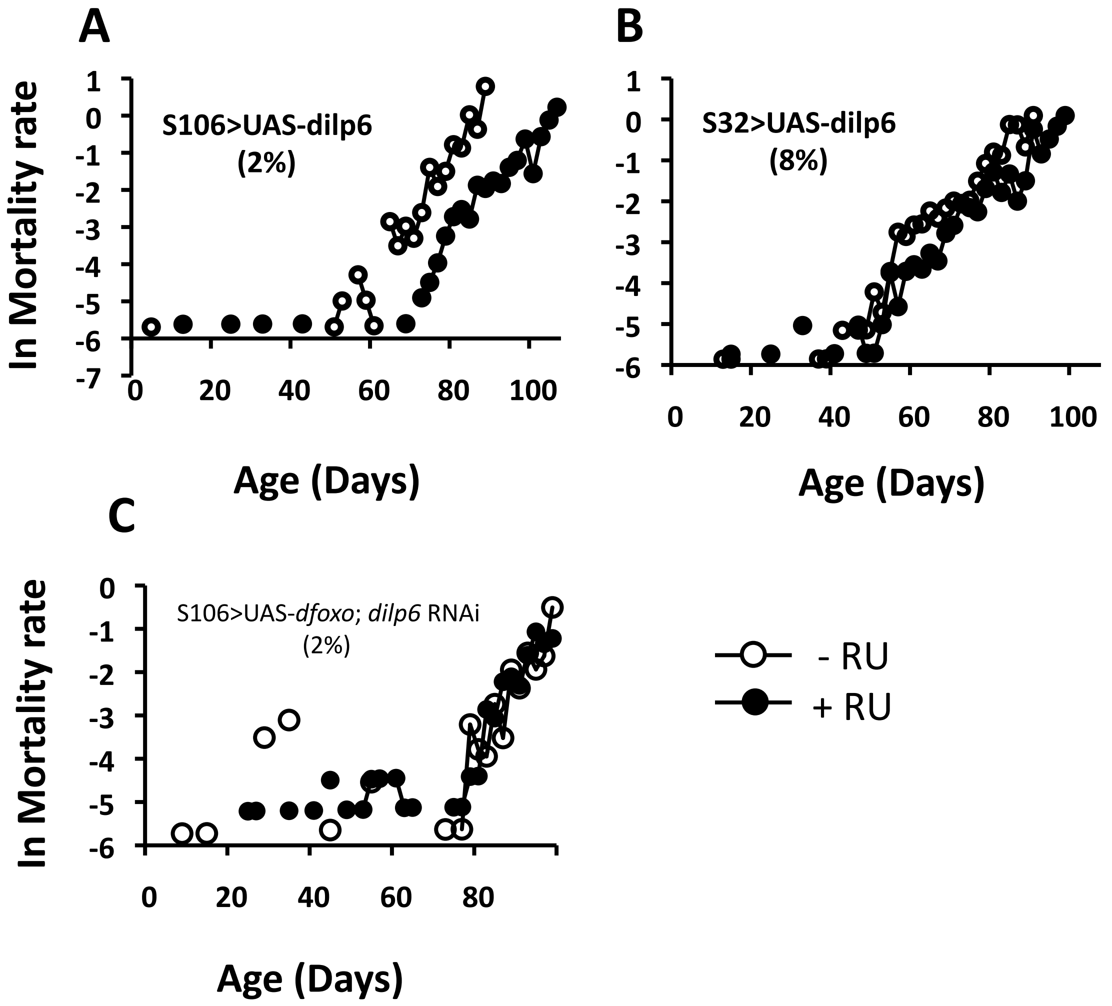

Supplement: Supplementary file 1 [file acel0011-0978-SD1.pdf]
