# Supplementary figures and images for: Drosophila insulin-like peptide-6 (dilp6) expression from fat body extends lifespan and represses secretion of Drosophila insulin-like peptide-2 from the brain
Source: Aging Cell. 2012 Dec;11(6):978–85. doi: 10.1111/acel.12000 (PMC3500397; doi:10.1111/acel.12000)

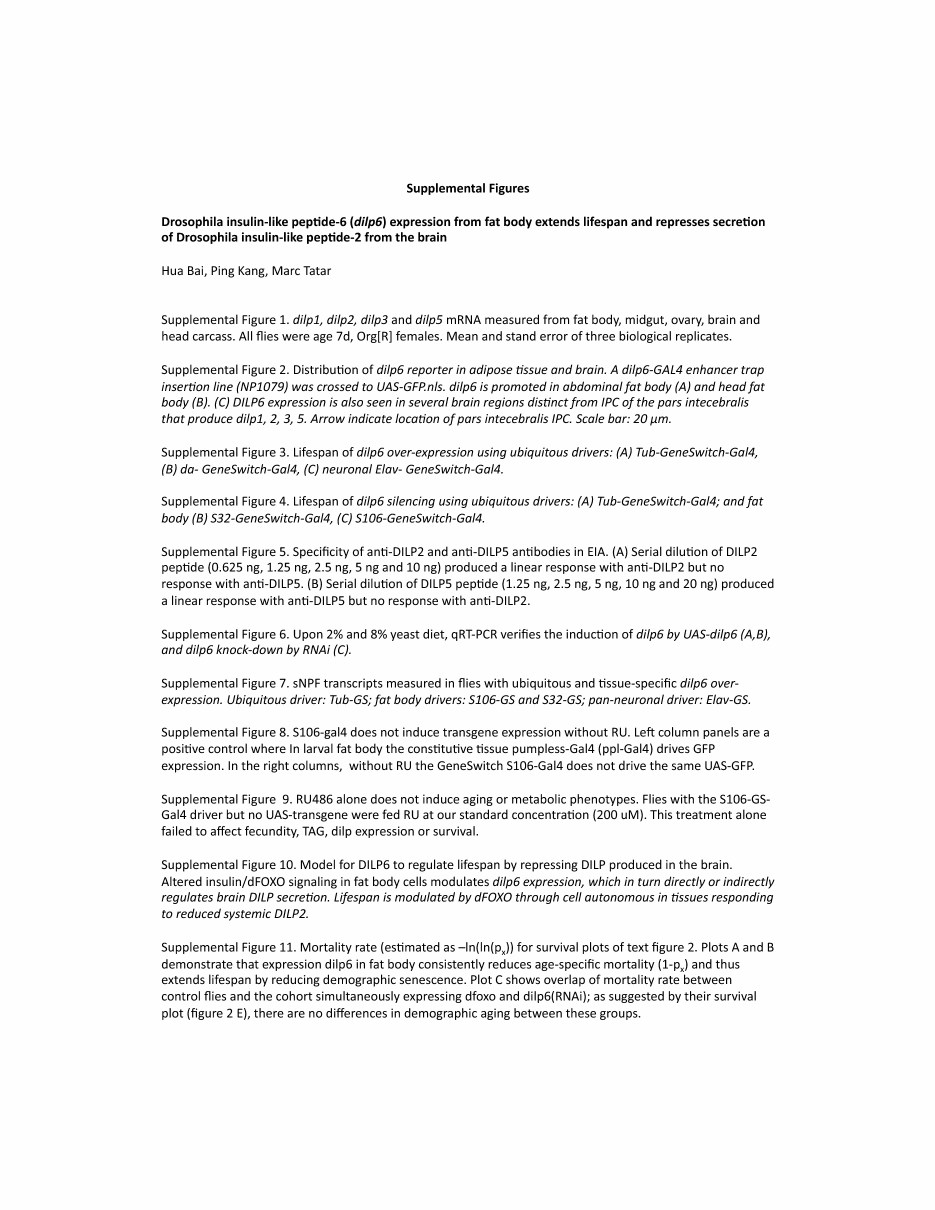

Supplement: Supplementary file 2 [file acel0011-0978-SD2.png]
